# The Impact of COVID-19 Pandemic on Orthopedic Trauma Practice: An Experience at a Tertiary Care Center in Northern India

**DOI:** 10.7759/cureus.20571

**Published:** 2021-12-21

**Authors:** Bhavkaran Singh, Rajesh Kapila, Kamalpreet Singh

**Affiliations:** 1 Orthopedics and Traumatology, Government Medical College, Amritsar, IND; 2 Orthopaedics, Government Medical College, Amritsar, IND

**Keywords:** pattern, trauma, orthopaedic, covid-19 pandemic, lockdown

## Abstract

Abstract: The Impact of Covid -19 Pandemic on Orthopedic trauma practice: An experience at a tertiary care center in Northern India

Introduction: COVID-19 pandemic and associated lockdown have had drastic effects on the health care system. To dedicate all the staff, residents, interns to covid care and accommodate the escalated inflow of infected persons, most hospitals had to restructure their departments. The orthopedic department was no exception. The present study aimed to evaluate trends in orthopedic trauma cases during two waves of the Covid -19 pandemic.

Material and Methods: In this retrospective observational study, the period of lockdown during the first wave of Covid (March 24, 2020, to May 31, 2020 (Period 2)) and the second wave in 2021 was compared with a similar nine weeks interval in 2019 before COVID -19 (Period 1). Demographic details and epidemiological parameters of trauma were collected and compared.

Observations: The number of admissions declined from 8.2 admissions/day to 2.3/day and 2.71/day in periods 2 and 3, respectively. Roadside accidents in 73.37% of patients in period 1 reduced to 30.43% and 59 36.2% in period 2 and 3, respectively. After soft tissue injuries, fractures around the hip joint dominated the pattern of injury during the lockdown, while polytrauma significantly decreased compared to the pre-covid era. More than 80% of patients during lockdown were treated conservatively.

Conclusion: Evaluation of differences in injury patterns and method of treatment during distinctive situations arising due to the COVID-19 pandemic will help to judicially plan and formulate protocols for more effective management of patients if similar events arise again.

Keywords: Pandemic, Covid-19, Lockdown, orthopedic trauma, trends

## Introduction

The first case of novel coronavirus SARS-COV-2(COVID-19) was identified in the city of Wuhan, China, in December 2019 [1], and by the middle of March 2020, when the outbreak involved over 190 countries. WHO declared it as a pandemic and global health emergency [2]. Though the first case reported in India on January 30, 2020, in Kerala was an Indian student who had traveled back from Wuhan city [3], Punjab in northern India reported its first case on March 5, 2020 [4]. Following the unprecedented community spread of the disease, a nationwide lockdown was declared on March 24, 2020, to mitigate further viral transmission and flatten the curve of transmissions [5]. A sharp decline in COVID 19 cases observed in September 2020 onwards in India may be attributed to effective intervention and implementation of government policies, health care workers' dedication, and increased public awareness.

Unfortunately, a false sense of normalcy and triumph resulted in added lethal second wave in early March 2021. On April 15, 2021, the daily count rose to double its first peak value [6]. A sudden surge in the number of cases could be due to the double mutant variant of SARS-CoV-2(B.1.617 lineage), the negligent behavior of the public, or the relaxation of COVID prevention guidelines. 

COVID-19 pandemic and associated lockdown have had drastic effects on day to day routine of individuals and institutions, especially the health care system. To dedicate all the staff, residents, interns to covid care and accommodate the escalated inflow of infected persons, most hospitals had to restructure their departments. All wards were converted into covid care units. There was an absolute suspension of non-emergency services, including outpatient clinics and elective surgeries. Similar to other departments, the Covid-19 pandemic also affected the orthopedics department.

The present retrospective observational study aimed to evaluate the change in trends in the number and type of orthopedic trauma cases during two waves of the Covid -19 pandemic by comparing it with the cohort of patients who presented in 2019 during a similar time frame.

## Materials and methods

The present retrospective observational study was conducted in Guru Nanak Dev Hospital, Government Medical College, a tertiary care center in Amritsar in North India. The data was collected from the hospital records department and confirmed daily hospital admission and discharge records from emergency and wards. The study period was from March 24, 2020, the start of lockdown during the first wave till May 31, 2020 (Period 2). This was compared with a similar nine weeks interval in 2019 before COVID -19 infection (Period 1) and a similar period in 2021 during the second wave of COVID 19 illness (Period 3).The study criteria comprised all patients presenting in the orthopedics emergency with fresh injuries or referred for tertiary level management. Patients presenting for follow-up or referred from other departments in our institution were excluded from the study. Their demographics, including age, gender, residential address, epidemiological factors of trauma, including the anatomical location of the injury, mode of injury, the pattern of injury like fractures (open/closed), dislocations, soft tissue injuries, were collected. As the study was purely a database-based observational study and no identifiable patient data was reported, it did not require any formal institutional ethical approval.

Statistical Analysis: Statistical package for social sciences software for windows (SPSS) version 21, IBM Corp. NY, USA was used for analysis [7]. All the data was collected on a Microsoft Excel spreadsheet. The Chi-square test was performed in cross-table evaluation. Unpaired t-test and one-way ANOVA were used to compare the means of two or more groups. A P-value of <0.5 was considered statistically significant.

The study did not require any funding.

## Results

Analysis of the database of our hospital revealed that during the pre covid period (period 1) in 2019, out of 492 patients admitted in an orthopedic emergency, 355 (72.2%) were males, and 137 (27.8%) were females (table 1).

**Table 1:** Demographic profile of patients presenting with orthopedic trauma

	Period 1: 25 March - 23 May 2019 (60days)	Period 2: 25 March - 23 May 2020 (60days)	Period 3: 25 March - 31 May 2021 (60days)	P-Value
Total No of Patients	492(8.2/day)	138(2.3/day)	163(2.71/day)	0.000
Female	137(27.8%)	39(28.26%)	42(25.7%)	0.854
Males	355(72.2%)	99(71.74%)	121(74.2%)	
<18 years of age	51(10.36%)	8(5.79%)	11(6.74%)	0.398
18-60 years of age	376(76.4%)	109(78.9%)	129(79.1%)	
>60 years	65(13.2%)	21(15.2%)	23(14.1%)	
Rural /urban	304/188	101/37	119/44	0.005

The number of admissions drastically declined to 138 patients in period 2, of which 99 (71.74%) were males, and 39 (28.26%)were females, and 163 patients in period 3, of which 121 (74.2%) were males and 42 (25.7%) were females. A total number of 51 (10.36%) patients (below 18 years) were admitted during period 1 (0.85 admissions/day), which decreased to 8 (5.79%) patients(0.13 admissions/day) in periods 2 and 11 (6.74%) patients (0.18 admissions/day)in period 3, while the number of patients admitted after trauma in working-class age group (between 18-60 years) recorded in all the three periods were similar. There were 376 (76.4%) (6.2 admissions/day) patients in period 1,109 (78.9%) patients in period 2 and 129 (79.1%) patients in period 3. During period 1, the number of elderly patients (age>60 years ) admitted after trauma were 65 (13.2%), which decreased to 21 (15.2%) and 23 (14.1%) in periods 2 and 3.

The main mode of injury observed during period 1 was roadside accidents in 361 (73.37%) patients, while it was slip and fall during period 2 in 56 (40.6%) patients and in 58 (35.6%) patients during period 3. There was a 43% reduction in roadside accident-related orthopedic trauma in period 2 and a 37.17% reduction in period 3 compared to period 1. Machine-related injuries decreased by 5.4% in period 2 and 5.46% in period 3 while injuries due to falling from height increased by 10.94 % in period 2 and 9.84% in period 3. These comparisons were statistically significant (Table 2).

**Table 2:** Comparison of Mode of Injury during Lockdown versus Non-lockdown periods.

Mode of injury	Period 1: 25 March - 31 May 2019	Period 2: 25 March - 31 May 2020	Period 3: 25 March - 31 May 2021	P-value
Road Traffic Accident	361(73.37%)	42(30.43%)	59(36.2%)	0.000
Fall From Height	21(4.26%)	21(15.2%)	23(14.1%)	0.000
Slip And Fall	53(10.77%)	56(40.6%)	58(35.6%)	0.000
Machine Injury	34(6.9%)	2(1.44%)	4(2.45%)	0.008
Fire injury /blast	10(2%)	5(3.6%)	7(4.3%)	0.250
Gun shot	3(0.6%)	2(1.44%)	1(0.61%)	0.586
Domestic violence/Assault	8(1.6%)	8(5.79%)	11(6.74%)	0.002
Sword injury	2(0.4%)	2(1.44%)	0(0%)	0.173
	492	138	163	

The commonest fracture encountered during lockdown was around the hip joint. (21.73% in 2020 and 20.2% in 2021).The rise was 14.7% and 13.9% in 2020 and 2021, respectively, as compared to in 2019, while a decline of 5.7% was reported in polytrauma during period 2 and 2.82% in period 3 compared to period 1 (Table 3).

**Table 3:** Comparison of Pattern of Injury during Lockdown versus non-lockdown periods.

Injury pattern	Period 1: 25 March - 31 May 2019 (492)	Period 2: 25 March - 31 May 2020 (138)	Period 3: 25 March - 31 May 2021 (163)	P-Value
Fracture around knee	12(2.4%)	4(2.8%)	4(2.4%)	0.953
Fracture around hip	32(6.5%)	30(21.73%)	33(20.2%)	0.000
Fracture around wrist	16(3.2%)	6(4.34%)	7(4.29%)	0.739
Fracture around ankle and foot	24(4.8%)	2(1.4%)	3(1.8%)	0.063
Fracture around shoulder	16(3.25%)	1(0.72%)	0(0%)	0.020
Fracture around elbow	6(1.2%)	4(2.9%)	4(2.4%)	0.315
Lower limb longbone fracture	108(21.95%)	5(3.62%)	12(7.36%)	0.000
Upper limb longbone fracture	28(5.69%)	12(8.69%)	15(9.20%)	0.073
Hand injury	55(11.17%)	4(2.9%)	4(2.4%)	0.000
Hip dislocation	0(0%)	0(0%)	1(0.61%)	0.538
Knee dislocation	0(0%)	0(0%)	0(0%)	-
Shoulder dislocation	9(1.8%)	8(5.8%)	9(5.52%)	0.014
Polytrauma	34(6.91%)	2(1.4%)	6(3.68%)	0.027
Spine injuries	42(8.5%)	15(10.86%)	20(12.26%)	0.332
Elbow dislocation	3(0.6%)	2(1.44%)	2(1.22%)	0.564
soft tissue injuries	176(35.77%)	47(34.05%)	55(33.7%)	0.846
Open/closed	201/291 40.8% / 59.2%	53/85 38.4%/61.6%	62/101 38% /62%	
	492	138	163	

A significant difference in interval between the time of injury and presentation to the emergency room was observed during the lockdown and non-covid period. The rate of conservative management drastically increased from 42.47% in period 1 to 87.68% and 80.98% in periods 2 and 3, respectively. The infection rate increased from 18.50% in period 1 to 24.63% and 23.31% in period 2 and 3, respectively (Table 4).

**Table 4:** Comparison of the mode of treatment during Lockdown versus non-Lockdown Periods

	Period 1	Period 2	Period 3	P- value
Open/closed	201/291 40.8% / 59.2%	53/85 38.4%/61.6%	62/101 38% /62%	0.759
Mean interval between trauma and presentation (days)	0.5 +/- 2.5	4.5+/-3.25	3.2+/-2.5	0.000
Secondary infection rate	91(18.5)%	34(24.63%)	38(23.31%)	0.179
Conservative treatment	199(42.47%)	121(87.68%)	132(80.98%)	0.000
Only Drugs	73(14.8%)	42(30.4%)	48(29.44%)	0.000
POP Cast/Slab/Traction	129(26.2%)	76(55.04%)	84(51.53%)	0.000
Intervention	293(57.53%)	17(12.31%)	31(19.01%)	
Soft tissue repair	103(20.93%)	5(3.62%)	7(4.29%)	0.000
Plating	78(15.8%)	7(5.07%)	9(5.52%)	0.000
Nailing	49((9.95%)	4(2.89%)	10(6.13%)	0.097
Kwire/screw fixation	38(7.7%)	2(1.44%)	6(3.68%)	0.040
Joint Replacement /External fixator	54(10.97%)	3(2.17%)	5(3.06%)	0.000
Amputation	3(0.60%)	1(0.72%)	0(0%)	0.079
	492	138	163	

## Discussion

COVID-19 pandemic had an enormous and unpredictable impact on various domains of life. The health care system was no exception. Workload related to COVID care increased boundlessly, and the whole system had to be revamped. During the pandemic, social distance restrictions and lockdown severely affected both outpatient and inpatient care.

In response to the guidelines issued by the Ministry of Health and Family Welfare(MoHFW), our tertiary care teaching hospital was designated as a COVID care center in this region. All the departments were reshuffled, and staff, residents, and interns from all specialties were pooled in for COVID duties. Given the shortage of staff and also to minimize cross-infection amongst patients and health care workers, all elective surgeries in our orthopedics department were postponed, routine outdoor patient clinics were closed, and only emergency and essential services were continued.

In this study, we evaluated and compared the effect of state-wide stay-at-home directives on orthopedic trauma during the COVID-19 pandemic in 2020 and 2021 with a calendar matched period in 2019.

In our region, there was a 72% reduction in the number of patients presenting with trauma during the first part of hard lockdown in 2020 (period 2), and to 66 .8% reduction is not such a hard lockdown during the second wave (period 3) when compared to corresponding time in 2019 (period 1). It was similar to the findings observed worldwide [8,9]. The reason might be the complete shutdown on roads and the non-availability of intoxicants, especially alcohol. The most typical age group affected during all three periods was the same (18-60 years), but a decline of 4.57 % was observed in patients below 18 years of age in period 2 and 3.62 % in period 3. This change might be because schools, colleges, and universities were closed, and all students were bound to the computers for online teaching and learning. An absolute number of male and female trauma-affected patients decreased during a lockdown, but the ratio was comparable in all periods.

Approximately 73% of trauma cases presenting in an emergency in period 1 were following motor-vehicle accidents (MVA). In comparison, due to extreme restrictions on leisure activities and movement of vehicles on the roads during a lockdown, these declined to 30.4% and 36.2% in periods 2 and 3, respectively. However, there was a significant increase in trauma due to slip and fall in period 2(30 %) and period 3(25%). It may be because of being confined to home. People were engaged in household activities like cleaning, which involved climbing stairs or rooftops for terrace gardening, playing, and other activities. During the pandemic, Lubbe RJ et al. also observed a decrease in orthopedic trauma consults, increased injuries due to gunshot wounds, and decreased automobile versus pedestrian accidents [8].

There was no difference in open and closed wounds between all periods under study, but fractures around the hip joint(21% in 2020 and 20.2% in 2021) accounted for most of the fractures during the lockdown. It was probably because the predominant mechanism of injury was slip and fall during this time. It increased by 15% and 14% in periods 2 and 3 compared to period 1. It was similar to the observations by surgeons in Jordan [9].

Similar to the observations in the literature interval between the time of injury and presentation to the emergency room significantly increased during the lockdown period compared to the non-covid period [8]. It could be due to lack of resources, travel restrictions, non-availability of transportation, and finally, because of fear of acquiring covid infection in the hospital, patients preferred to stay home rather than being treated in our hospital filled with COVID -19 infected patients.

Though there was a significant decrease in the total number of open and closed fractures during the lockdown, the rate of conservative management drastically increased from 42.47% in period 1 to 87.68% and 80.98% in the 2nd and 3rd period respectively. There was a universal acceptance that it was better to accept sub-optimal post-traumatic outcomes rather than acquiring COVID-19 infection during their hospital stay. Moreover, orthopedic procedures require the use of drills and saws that are aerosol-generating, so only life-threatening or limb-saving surgeries were performed during this period. Similar observations were recorded in other parts of the world [9-11].

The infection rate increased from 18.50% in period 1 to 24.63% and 23.31% in periods 2 and 3, respectively. It could be because of the delay in presentation to the orthopedic emergency and the increased time interval between presentation and the management due to the implementation of covid protocols. The observations of Ketonis et al. recorded a correlation between early intervention and administration of antibiotics with decreased infection rate. In contrast, their study found no statistical difference in infection rates when early debridement was done [12,13].

## Conclusions

COVID-19 pandemic and subsequent lockdown in India observed a noticeable decline in orthopedic trauma patients. There was significant variation in pattern and mechanism of injury, and the paradigm shifted towards conservative management. Evaluation of differences in injury patterns during distinctive situations arising due to the COVID-19 pandemic is required to judicially plan and formulate protocols for more effective management of patients if similar events arise again.
